# Supplementary material for: Associations between Smoking and Smoking Cessation during Pregnancy and Newborn Metabolite Concentrations: Findings from PRAMS and INSPIRE Birth Cohorts
Source: Metabolites. 2023 Nov 19;13(11):1163. doi: 10.3390/metabo13111163 (PMC10673147; doi:10.3390/metabo13111163)
Supplement: Supplementary file 1 [file metabolites-13-01163-s001.zip › metabolites-2649546-supplementary.pdf]

Associations between Smoking and Smoking Cessation during Pregnancy and Newborn Metabolite Concentrations: Findings from PRAMS and INSPIRE Birth Cohorts

Brittney M. Snyder, Hui Nian, Angela M. Miller, Kelli K. Ryckman, Yinmei Li, Hilary A. Tindle, Lin Ammar, Abhismitha Ramesh, Zhouwen Liu, Tina V. Hartert, Pingsheng Wu

Supplementary Material

Methods and materials 2
Newborn screening metabolic data collection 2
Covariate ascertainment 2
References 3
Table S1 5
Table S2. 6
Figure S1 7
Figure S2 8
Figure S3 9
Figure S4. 10
Figure S5. 11
Table S3 12
Table S4 15
Table S5 17
Table S6 22
Figure S6 25
Table S7 26
Figure S7. 27
Figure S8 28

Methods and materials

Newborn screening metabolic data collection

Our primary outcomes were concentrations of 33 targeted metabolites at birth ascertained from the Tennessee NBS metabolic panel and provided by the Tennessee Department of Health NBS program. Tennessee NBS metabolic data include targeted measurement of free carnitine, 21 acylcarnitines, and 11 amino acids (**Table S2**). NBS is a public health initiative aimed at preventing adverse health effects associated with inborn errors of metabolism [1]. Standardized collection of blood spot cards was performed by a healthcare professional 24–48 hours after birth. Cards were then sent to the state laboratory for routine testing [1,2]. Tandem mass spectrometry was used to quantitatively measure metabolite concentrations using the calculated ratio of the signal from each metabolite to the signal from the known amount of internal standard [3]. Quantified results were stored on the Tennessee Department of Health server [2]. Data were curated according to our previously established protocol [2,4,5].

Covariate ascertainment

We selected covariates based on clinical relevance or published evidence of their association with prenatal smoking or infant metabolism. Maternal characteristics included maternal age at delivery (<20, 20-24, 25-29, 30-34, ≥35 years), pre-pregnancy body mass index (<18.5, 18.5-24.9, 25.0-29.9, ≥30.0), maternal race and ethnicity (Non-Hispanic White; Non-Hispanic Black; Hispanic; Other [including Non-Hispanic Asian, Non-Hispanic American Indian, Non-Hispanic Chinese, Non-Hispanic Japanese, Non-Hispanic Filipino, Non-Hispanic mixed race, and Non-Hispanic other race for PRAMS and Non-Hispanic Asian, Non-Hispanic Hawaiian, Non-Hispanic multiple race, and Non-Hispanic Native American for INSPIRE]),

education (<12, 12, 13-15, ≥16 years), marital status (married, other [including single and separated/divorced for INSPIRE]), delivery method (vaginal, cesarean section), type of health insurance (government, private, other [including self-pay, Indian Health System, and 'other' categories for PRAMS and self-pay and 'other' categories for INSPIRE]), residence (urban, rural), pregnancy weight gain (lbs, continuous), pregnancy hypertension (yes, no), and gestational diabetes (yes, no). Infant characteristics included gender (female, male), birth weight (grams, continuous), gestational age (weeks, continuous), ever breastfed (yes, no), and birth year (continuous). For PRAMS, covariates were ascertained from linked birth certificates. For INSPIRE, all covariates, excluding maternal race and ethnicity, were ascertained from enrollment questionnaires. Maternal race and ethnicity were ascertained from questionnaires administered when the child reached the age of six. For infants with missing maternal race and ethnicity (n=768 [40%]), infant race and ethnicity was used as a proxy variable (kappa 0.66).

References

1. National Institutes of Health. Newborn Screening. Available online: https://www.nichd.nih.gov/health/topics/newborn (accessed on 20 September 2023).
2. Snyder, B.M.; Gebretsadik, T.; Rohrig, N.B.; Wu, P.; Dupont, W.D.; Dabelea, D.M.; Fry, R.C.; Lynch, S.V.; McEvoy, C.T.; Paneth, N.S.; et al. The Associations of Maternal Health Characteristics, Newborn Metabolite Concentrations, and Child Body Mass Index among US Children in the ECHO Program. *Metabolites* **2023**, *13*, doi:10.3390/metabo13040510.
3. CLSI. Newborn Screening by Tandem Mass Spectrometry. 2nd ed. CLSI guideline NBS04. Wayne, PA: Clinical and Laboratory Standards Institute; 2017.

4. Donovan, B.M.; Ryckman, K.K.; Breheny, P.J.; Gebretsadik, T.; Turi, K.N.; Larkin, E.K.; Li, Y.; Dorley, M.C.; Hartert, T.V. Association of newborn screening metabolites with risk of wheezing in childhood. *Pediatr Res* **2018**, *84*, 619-624, doi:10.1038/s41390-018-0070-4.
5. Snyder, B.M.; Gebretsadik, T.; Turi, K.N.; McKennan, C.; Havstad, S.; Jackson, D.J.; Ober, C.; Lynch, S.; McCauley, K.; Seroogy, C.M.; et al. Association of citrulline concentration at birth with lower respiratory tract infection in infancy: Findings from a multi-site birth cohort study. *Front Pediatr* **2022**, *10*, 979777, doi:10.3389/fped.2022.979777.

Table S1. Ascertainment of perinatal smoking by cohort.

PRAMS		
**Ascertainment source**	**Time frame**	**Description**
Birth certificate	Three months prior to pregnancy	Women reported the number of cigarettes smoked per day during the respective time period. Zero indicated no smoking during the respective time period.
	First three months of pregnancy	
	Second three months of pregnancy	
	Last three months of pregnancy	
**INSPIRE**		
**Ascertainment source**	**Time frame**	**Description**
Enrollment questionnaire[a]	Year prior to pregnancy	Women reported smoking (yes/no) during the respective time period. If an affirmative response to smoking was reported, women were further asked about the number of cigarettes smoked per day during the respective time period.
	First trimester of pregnancy	
	Second trimester of pregnancy	
	Third trimester of pregnancy	

[a]Enrollment questionnaire administered at infant age ~2 months.

Table S2. Metabolites ascertained from the Tennessee newborn screening panel.

Metabolite name (units)	Metabolite abbreviation
Free carnitine (umol/L)	C0
**Short-chain acylcarnitines (umol/L)**	
Acetylcarnitine	C2
Propionylcarnitine	C3
Butyrylcarnitine + Isobutyrylcarnitine	C4
Isovalerylcarnitine + Methylbutyrylcarnitine	C5
Tiglylcarnitine	C5:1
**Medium-chain acylcarnitines (umol/L)**	
Hexanoylcarnitine	C6
Methylglutarylcarnitine	C6-DC
Octanoylcarnitine	C8
Decanoylcarnitine	C10
Decenoylcarnitine	C10:1
Decadienoylcarnitine	C10:2
**Long-chain acylcarnitines (umol/L)**	
Tetradecanoylcarnitine	C14
Tetradecenoylcarnitine	C14:1
3-Hydroxytetradecanoylcarnitine	C14-OH
Palmitoylcarnitine	C16
Palmitoleylcarnitine	C16:1
3-Hydroxypalmitoylcarnitine	C16-OH
Stearoylcarnitine	C18
Oleoylcarnitine	C18:1
Linoleoylcarnitine	C18:2
3-Hydroxyoleoylcarnitine	C18:1-OH
**Amino Acids (umol/L)**	
Arginine	ARG
Argininosuccinate	ASA
Citrulline	CIT
Glycine	GLY
Leucine	LEU
Methionine	MET
Ornithine	ORN
Phenylalanine	PHE
Succinylacetone	SUAC
Tyrosine	TYR
Valine	VAL

Figure S1. ***A priori*** **statistical analysis plan.**

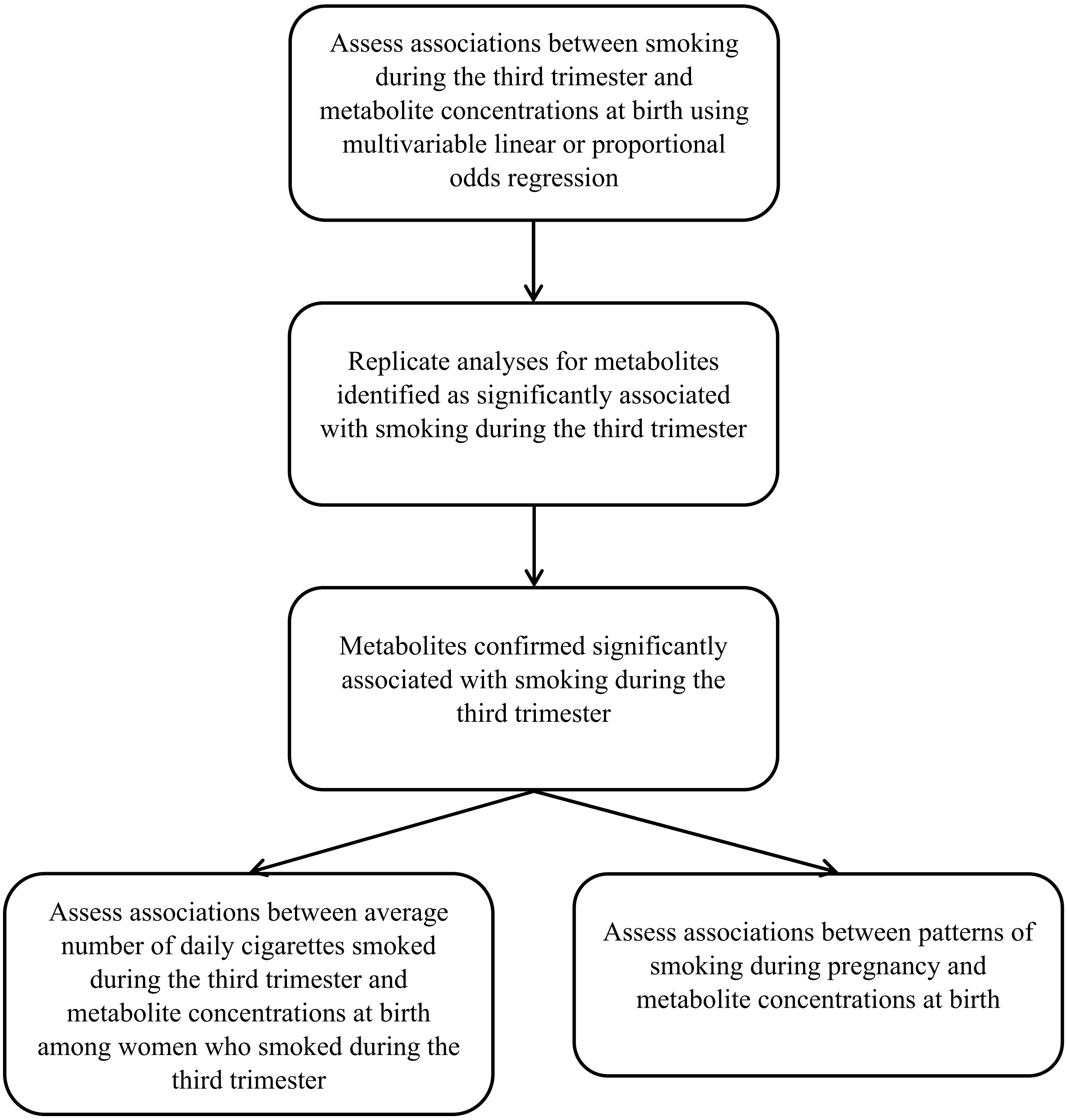

Figure S2. Distributions of A) continuous and B) ordinal* metabolite concentrations by cohort.

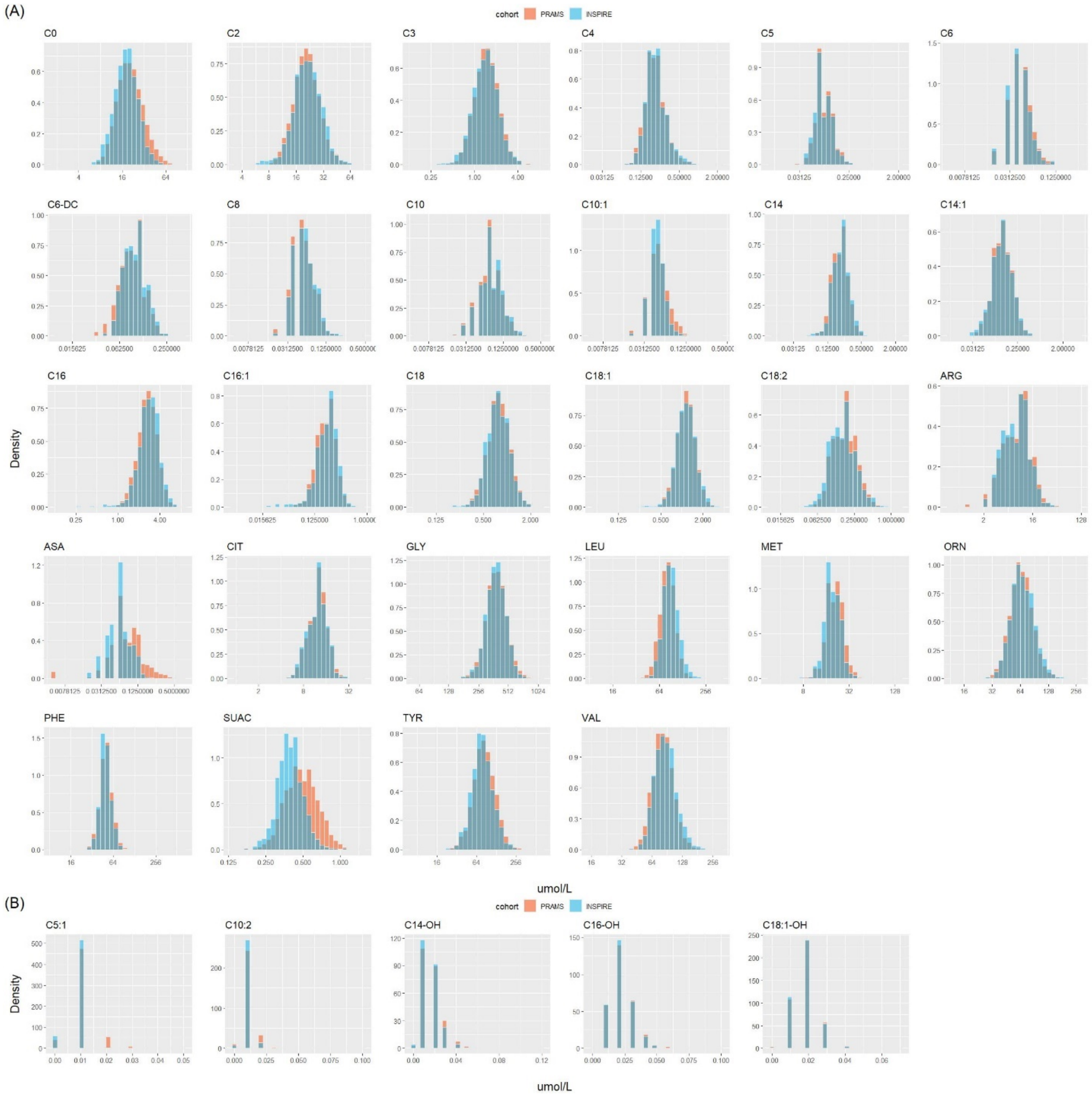

*Ordinal metabolites were defined as having <15 unique values.

Figure S3. Directed acyclic graph of associations between smoking during the third trimester of pregnancy and metabolite concentrations at birth.

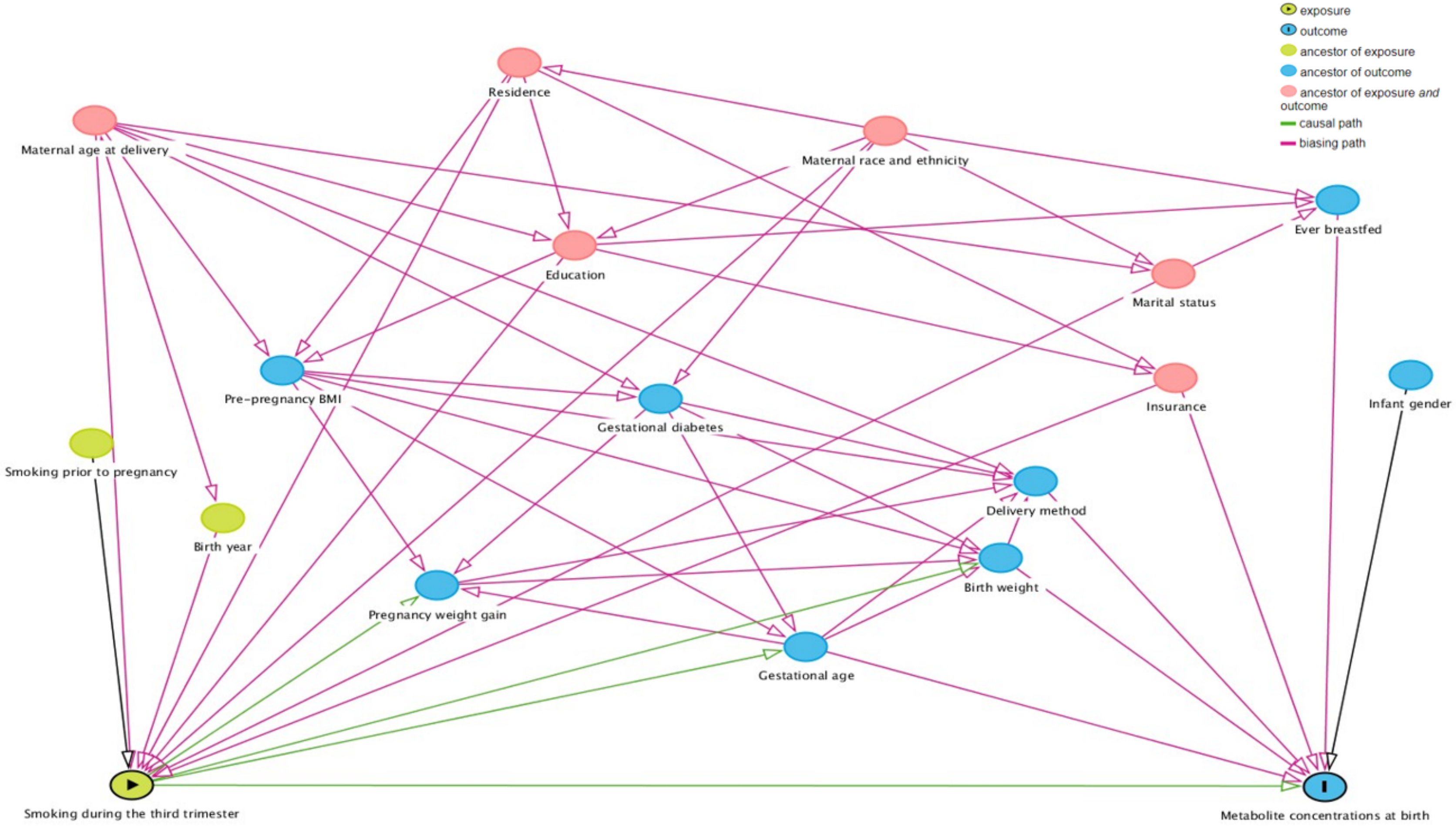

Figure created with DAGitty (www.dagitty.net).

Figure S4. Distribution of number of cigarettes smoked per day during the third trimester among women who smoked during the third trimester by cohort (PRAMS: n=1218; INSPIRE: n=240).

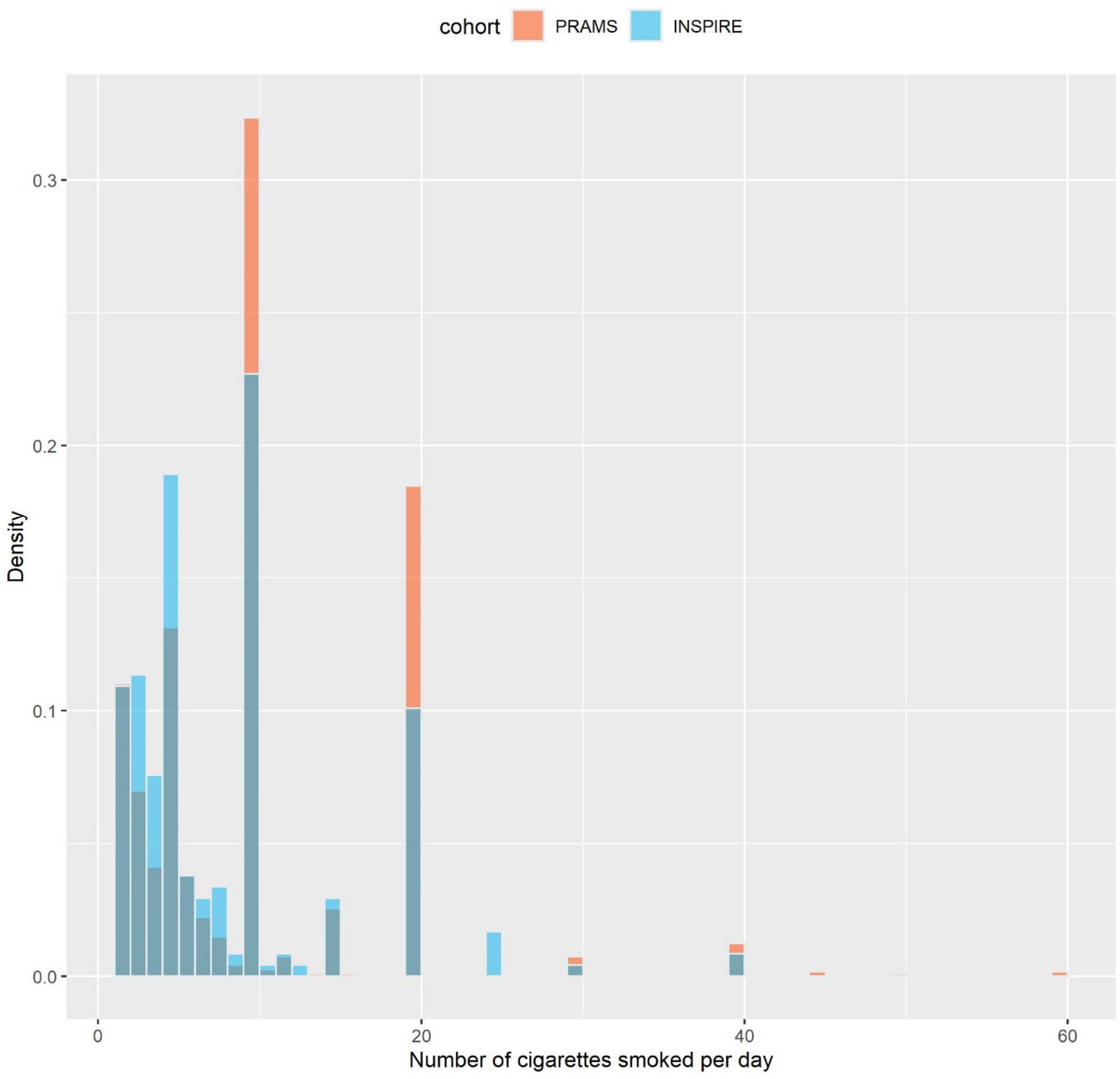

Figure S5. Lasagna plots of smoking status over time among A) PRAMS and B) INSPIRE women.

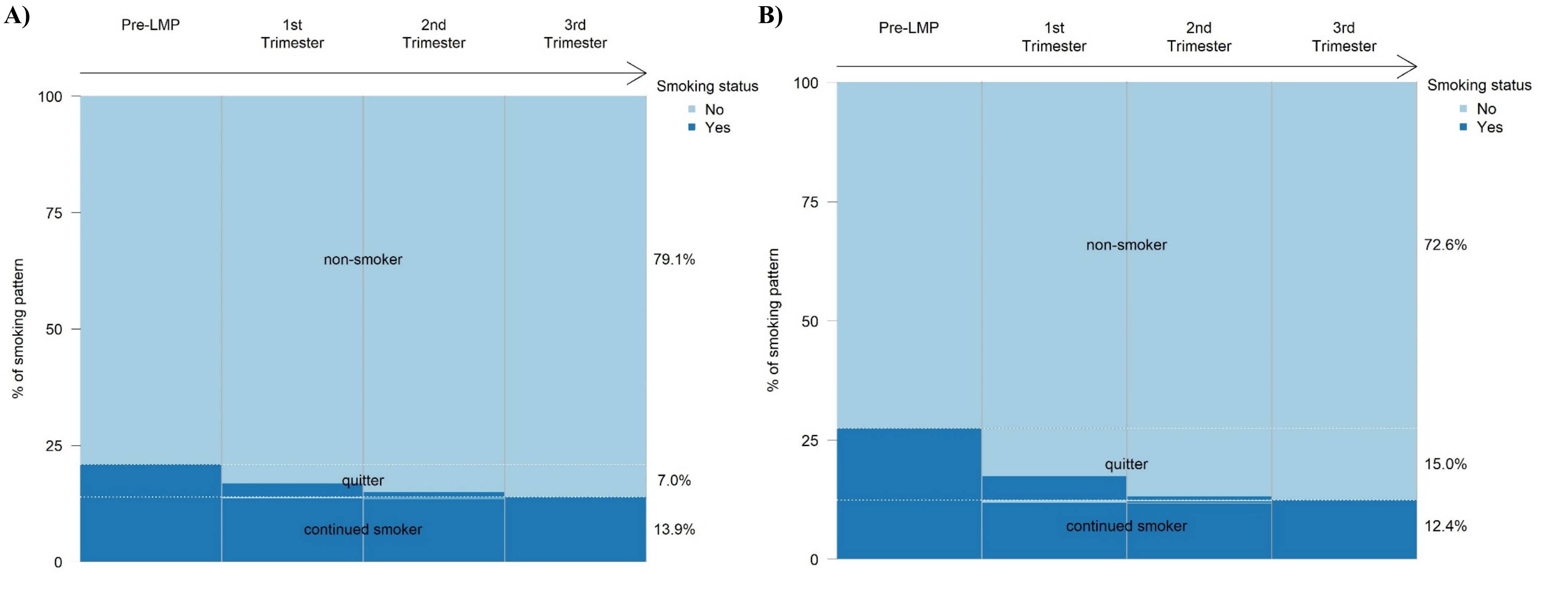

LMP, last menstrual period.

Each woman is depicted as a horizontal line. Light and dark blue horizontal lines indicate no smoking and smoking during the respective time period.

Table S3. Maternal characteristics by third-trimester smoking status of the study populations with linked newborn screening metabolic data.

	PRAMS			INSPIRE		
**Maternal characteristic**	**Smoking during third trimester**	**No smoking during third trimester**	***p*-value[a]**	**Smoking during third trimester**	**No smoking during third trimester**	***p*-value[a]**
Sample size, n (%)	1218 (14)	7382 (86)		240 (13)	1678 (87)	
Race and ethnicity, n (%)			<0.001*			0.006*
Non-Hispanic White	1019 (84)	4429 (60)		167 (70)	1079 (64)	
Non-Hispanic Black	159 (13)	1769 (24)		33 (14)	307 (18)	
Hispanic	15 (1)	782 (11)		11 (5)	155 (9)	
Other[b]	25 (2)	385 (5)		29 (12)	137 (8)	
Missing, n (%)	0 (0)	17 (0)		0 (0)	0 (0)	
Education (years), n (%)			<0.001*			<0.001*
<12	342 (28)	1138 (15)		63 (26)	89 (5)	
12	567 (47)	1983 (27)		101 (42)	422 (25)	
13-15	284 (23)	2265 (31)		65 (27)	508 (30)	
≥16	25 (2)	1970 (27)		10 (4)	659 (39)	
Missing, n (%)	0 (0)	26 (0)		1 (0)	0 (0)	
Marital status, n (%)			<0.001*			<0.001*
Married	395 (32)	4143 (56)		73 (30)	1029 (61)	
Other[c]	823 (68)	3239 (44)		167 (70)	649 (39)	
Missing, n (%)	0 (0)	0 (0)		0 (0)	0 (0)	
Age at delivery (years), n (%)			<0.001*			<0.001*
<20	160 (13)	721 (10)		20 (8)	129 (8)	
20-24	430 (35)	1884 (26)		94 (39)	454 (27)	
25-29	356 (29)	2177 (29)		71 (30)	484 (29)	
30-34	195 (16)	1692 (23)		42 (18)	445 (27)	
≥35	77 (6)	908 (12)		13 (5)	166 (10)	
Missing, n (%)	0 (0)	0 (0)		0 (0)	0 (0)	

Delivery method, n (%)			0.01*			0.04*
Vaginal	838 (69)	5340 (72)		151(63)	1167 (70)	
Cesarean section	380 (31)	2042 (28)		89 (37)	511 (30)	
Missing, n (%)	0 (0)	0 (0)		0 (0)	0 (0)	
Insurance, n (%)			<0.001*			<0.001*
Government	984 (81)	3541 (48)		206 (86)	835 (50)	
Private	201 (17)	3345 (45)		33 (14)	822 (49)	
Other[d]	15 (1)	202 (3)		--	19 (1)	
Missing, n (%)	18 (1)	294 (4)		--	2 (0)	
Residence, n (%)			<0.001*			<0.001*
Urban	344 (28)	3466 (47)		144 (60)	1304 (78)	
Rural	733 (60)	3220 (44)		92 (38)	361 (22)	
Missing, n (%)	141 (12)	696 (9)		4 (2)	13 (1)	
Pre-pregnancy BMI, n (%)			<0.001*			0.12
<18.5	256 (21)	1811 (25)		67 (28)	399 (24)	
18.5-24.9	262 (22)	1749 (24)		53 (22)	415 (25)	
25.0-29.9	542 (44)	3301 (45)		93 (39)	753 (45)	
≥30.0	144 (12)	316 (4)		12 (5)	54 (3)	
Missing, n (%)	14 (1)	205 (3)		15 (6)	57 (3)	
Pregnancy weight gain (kgs), median (IQR)	14 (9-19)	14 (10-18)	0.19	14 (9-20)	15 (10-19)	0.83
Pregnancy weight gain (lbs), median (IQR)	30 (20-42)	31 (21-40)	0.19	31 (20-45)	32 (23-42)	0.83
Missing, n (%)	46 (4)	351 (5)		18 (8)	68 (4)	
Pregnancy hypertension, n (%)	77 (6)	574 (8)	0.08	26 (11)	127 (8)	0.13
Missing, n (%)	0 (0)	0 (0)		87 (36)	668 (40)	
Gestational diabetes, n (%)	59 (5)	399 (5)	0.42	17 (7)	109 (6)	0.73
Missing, n (%)	0 (0)	0 (0)		0 (0)	0 (0)	

BMI—body mass index; IQR—interquartile range.
[a]*p*-values calculated using Mann–Whitney U or Pearson χ^2, as appropriate.

[b]'Other' category included Non-Hispanic Asian, Non-Hispanic American Indian, Non-Hispanic Chinese, Non-Hispanic Japanese, Non-Hispanic Filipino, Non-Hispanic mixed race, and Non-Hispanic other race for PRAMS and Non-Hispanic Asian, Non-Hispanic Hawaiian, Non-Hispanic multiple race, and Non-Hispanic Native American for INSPIRE.
[c]'Other' category included single and separated/divorced for INSPIRE.
[d]'Other' category included self-pay, Indian Health System, and 'other' categories for PRAMS and self-pay and 'other' categories for INSPIRE.
*Statistically significant at $\alpha<0.05$.
--, Data suppressed ($n<10$).

Table S4. Infant characteristics by third-trimester smoking status of the study populations with linked newborn screening metabolic data.

Infant characteristic	PRAMS			INSPIRE		
	Smoking during third trimester	No smoking during third trimester	*p*-value[a]	Smoking during third trimester	No smoking during third trimester	*p*-value[a]
Sample size, n (%)	1218 (14)	7382 (86)		240 (13)	1678 (87)	
Gender, n (%)			0.05			0.58
Female	651 (53)	3718 (50)		110 (46)	801 (48)	
Male	567 (47)	3664 (50)		130 (54)	877 (52)	
Missing, n (%)	0 (0)	0 (0)		0 (0)	0 (0)	
Birth weight (grams), median (IQR)	3,260 (2828-3550)	3,260 (2835-3572)	0.99	3,235 (2923-3547)	3433 (3150-3746)	<0.001*
Missing, n (%)	0 (0)	0 (0)		0 (0)	0 (0)	
Gestational age (weeks), median (IQR)	39 (38-39)	39 (38-39)	<0.001*	39 (38-40)	39 (39-40)	0.01*
Missing, n (%)	0 (0)	0 (0)		0 (0)	0 (0)	
Ever breastfed, n (%)	597 (49)	5533 (75)	<0.001*	129 (54)	1340 (80)	<0.001*
Missing, n (%)	31 (3)	294 (4)		0 (0)	0 (0)	
Birth year, n (%)			<0.001*			N/A
2009	112 (9)	454 (6)		N/A	N/A	
2010	181 (15)	911 (12)		N/A	N/A	
2011	188 (15)	936 (13)		N/A	N/A	
2012	95 (8)	604 (8)		94 (39)	750 (45)	
2013	120 (10)	524 (7)		146 (61)	928 (55)	
2014	41 (3)	128 (2)		N/A	N/A	
2015	58 (5)	381 (5)		N/A	N/A	
2016	135 (11)	930 (13)		N/A	N/A	
2017	130 (11)	1005 (14)		N/A	N/A	
2018	97 (8)	967 (13)		N/A	N/A	
2019	61 (5)	542 (7)		N/A	N/A	
Missing, n (%)	0 (0)	0 (0)		0 (0)	0 (0)	

IQR, interquartile range; N/A, not applicable.

[a]p-values calculated using Mann–Whitney U or Pearson χ^2, as appropriate.
*Statistically significant at $\alpha<0.05$.

Table S5. Newborn screening metabolite concentrations by third-trimester smoking status and cohort.

Metabolite	PRAMS			INSPIRE		
	Smoking during third trimester	No smoking during third trimester	*p*-value[a]	Smoking during third trimester	No smoking during third trimester	*p*-value[a]
**Sample size, n (%)**	1218 (14)	7382 (86)		240 (13)	1678 (87)	
Free carnitine (C0), median (IQR)	22.62 (17.27-30.65)	19.89 (15.19-26.45)	<0.001*	20.31 (15.92-25.20)	17.83 (13.89-22.81)	<0.001*
Missing, n (%)	2 (0)	18 (0)		2 (1)	6 (0)	
**Short-chain acylcarnitines**						
Acetylcarnitine (C2), median (IQR)	21.45 (17.11-27.00)	20.50 (16.60-25.67)	<0.001*	22.11 (17.46-27.12)	21.08 (16.90-27.00)	0.40
Missing, n (%)	2 (0)	25 (0)		2 (1)	6 (0)	
Propionylcarnitine (C3), median (IQR)	1.50 (1.18-1.92)	1.45 (1.13-1.89)	0.008*	1.47 (1.17-1.90)	1.42 (1.08-1.85)	0.16
Missing, n (%)	2 (0)	24 (0)		2 (1)	6 (0)	
Butyrylcarnitine + Isobutyrylcarnitine (C4), median (IQR)	0.20 (0.16-0.26)	0.21 (0.16-0.26)	0.73	0.20 (0.16-0.25)	0.21 (0.17-0.26)	0.22
Missing, n (%)	1 (0)	23 (0)		2 (1)	6 (0)	
Isovalerylcarnitine + Methylbutyrylcarnitine (C5), median (IQR)	0.09 (0.07-0.12)	.09 (0.07-0.11)	<0.001*	0.09 (0.07-0.11)	0.08 (0.07-0.11)	0.04*
Missing, n (%)	2 (0)	30 (0)		2 (1)	11 (1)	
Tiglylcarnitine (C5:1), n (%)			0.005*			0.92
0	98 (8)	489 (7)		26 (11)	172 (10)	
0.01	969 (80)	6053 (82)		211 (88)	1487 (89)	
0.02	137 (11)	684 (9)		--	12 (1)	
≥0.03	12 (1)	138 (2)		--	--	
Missing, n (%)	2 (0)	18 (0)		--	--	
**Medium-chain acylcarnitines**						
Hexanoylcarnitine (C6), median (IQR)	0.05 (0.04-0.06)	0.05 (0.04-0.06)	0.31	0.04 (0.03-0.05)	0.04 (0.04-0.06)	0.61

Missing, n (%)	2 (0)	20 (0)		2 (1)	6 (0)	
Methylglutarylcarnitine (C6-DC), median (IQR)	0.09 (0.07-0.12)	0.09 (0.07-0.12)	0.16	0.10 (0.08-0.12)	0.10 (0.08-0.12)	0.78
Missing, n (%)	2 (0)	31 (0)		2 (1)	8 (0)	
Octanoylcarnitine (C8), median (IQR)	0.05 (0.04-0.07)	0.06 (0.04-0.07)	0.06	0.05 (0.04-0.07)	0.06 (0.05-0.07)	0.005*
Missing, n (%)	1 (0)	16 (0)		2 (1)	6 (0)	
Decanoylcarnitine (C10), median (IQR)	0.07 (0.05-0.10)	0.08 (0.06-0.10)	<0.001*	0.07 (0.05-0.10)	0.08 (0.06-0.11)	<0.001*
Missing, n (%)	3 (0)	17 (0)		2 (1)	6 (0)	
Decenoylcarnitine (C10:1), median (IQR)	0.05 (0.04-0.07)	0.05 (0.04-0.07)	0.46	0.05 (0.04-0.06)	0.05 (0.04-0.06)	0.25
Missing, n (%)	2 (0)	16 (0)		2 (1)	6 (0)	
Decadienoylcarnitine (C10:2), n (%)			0.002*			0.88
0	43 (4)	277 (4)		--	41 (2)	
0.01	978 (80)	6202 (84)		220 (92)	1550 (92)	
0.02	179 (15)	816 (11)		12 (1)	77 (5)	
≥0.03	16 (1)	71 (1)		--	--	
Missing, n (%)	2 (0)	16 (0)		--	--	
**Long-chain acylcarnitines**						
Tetradecanoylcarnitine (C14), median (IQR)	0.20 (0.17-0.26)	0.21 (0.16-0.26)	0.43	0.21 (0.16-0.26)	0.22 (0.17-0.27)	0.03*
Missing, n (%)	2 (0)	20 (0)		2 (1)	6 (0)	
Tetradecenoylcarnitine (C14:1), median (IQR)	0.11 (0.08-0.15)	0.13 (0.09-0.17)	<0.001*	0.10 (0.08-0.14)	0.13 (0.09-0.17)	<0.001*
Missing, n (%)	2 (0)	22 (0)		2 (1)	6 (0)	
3-Hydroxytetrad- ecanoylcarnitine (C14-OH), n (%)			<0.001*			<0.001*
0	16 (1)	69 (1)		--	28 (2)	
0.01	639 (52)	3224 (44)		152 (63)	779 (46)	
0.02	373 (31)	2801 (38)		61 (25)	664 (40)	
0.03	135 (11)	935 (13)		18 (8)	164 (10)	

0.04	38 (3)	227 (3)		--	34 (2)	
≥0.05	15 (1)	104 (1)		--	--	
Missing, n (%)	2 (0)	22 (0)		--	--	
Palmitoylcarnitine (C16), median (IQR)	2.57 (2.09-3.19)	2.67 (2.16-3.30)	0.001*	2.63 (2.12-3.33)	2.83 (2.28-3.45)	0.004*
Missing. N (%)	2 (0)	24 (0)		2 (1)	6 (0)	
Palmitoleylcarnitine (C16:1), median (IQR)	0.19 (0.14-0.24)	0.20 (0.15-0.26)	<0.001*	0.19 (0.13-0.25)	0.21 (0.16-0.27)	<0.001*
Missing, n (%)	3 (0)	24 (0)		2 (1)	6 (0)	
3-Hydroxypalmi-toylcarnitine (C16-OH), n (%)			<0.001*			<0.001*
0	--	10 (0)		--	--	
0.01	313 (26)	1412 (19)		80 (33)	309 (18)	
0.02	559 (46)	3575 (48)		109 (45)	856 (51)	
0.03	251 (21)	1669 (23)		37 (15)	380 (23)	
0.04	71 (6)	487 (7)		10 (4)	97 (6)	
0.05	12 (1)	130 (2)		--	24 (1)	
0.06	--	50 (1)		--	--	
≥0.07	--	24 (0)		--	--	
Missing, n (%)	--	25 (0)		--	--	
Stearoylcarnitine (C18), median (IQR)	0.80 (0.64-1.00)	0.78 (0.63-0.96)	0.003*	0.78 (0.59-0.95)	0.77 (0.62-0.96)	0.60
Missing, n (%)	3 (0)	24 (0)		2 (1)	6 (0)	
Oleoylcarnitine (C18:1), median (IQR)	1.18 (0.96-1.44)	1.15 (0.94-1.39)	0.002*	1.15 (0.92-1.43)	1.16 (0.93-1.42)	0.83
Missing, n (%)	3 (0)	24 (0)		2 (1)	6 (0)	
Linoleoylcarnitine (C18:2), median (IQR)	0.22(0.16-0.29)	0.17 (0.12-0.23)	<0.001*	0.19 (0.14-0.26)	0.16 (0.12-0.22)	<0.001*
Missing, n (%)	3 (0)	26 (0)		2 (1)	6 (0)	
3-Hydroxyoleoylcarnitine (C18:1-OH), n (%)			0.11			0.98
0	11 (1)	48 (1)		--	--	
0.01	344 (28)	1899 (26)		68 (28)	459	

0.02	666 (55)	4268 (58)		134 (56)	969 (27)	
0.03	166 (14)	1024 (14)		32 (13)	219 (13)	
0.04	24 (2)	97 (1)		--	17 (1)	
0.05	--	15 (0)		--	--	
≥0.06	--	--		--	--	
Missing, n (%)	--	--		--	--	
**Amino Acids**						
Arginine (ARG), median (IQR)	8.00 (6.00-12.00)	9.00 (6.00-12.00)	0.18	8.00 (6.00-11.00)	8.00 (6.00-11.00)	0.83
Missing, n (%)	1 (0)	14 (0)		2 (1)	4 (0)	
Argininosuccinate (ASA), median (IQR)	0.08 (0.06-0.12)	0.08 (0.06-0.12)	0.37	0.07 (0.05-0.09)	0.06 (0.05-0.08)	0.15
Missing, n (%)	177 (15)	797 (11)		2 (1)	4 (0)	
Citrulline (CIT), median (IQR)	12.00 (10.00-14.00)	12.00 (10.00-15.00)	<0.001*	12.00 (10.00-14.00)	12.00 (10.00-15.00)	0.07
Missing, n (%)	3 (0)	15 (0)		2 (1)	4 (0)	
Glycine (GLY), median (IQR)	389.00 (327.50-462.00)	385.00 (329.00-450.00)	0.39	390.00 (338.25-446.00)	383.00 (329.00-443.75)	0.28
Missing, n (%)	3 (0)	15 (0)		2 (1)	4 (0)	
Leucine (LEU), median (IQR)	86.00 (73.00-100.00)	81.00 (70.00-94.00)	<0.001*	94.00 (83.00-108.75)	89.00 (76.00-103.75)	<0.001*
Missing, n (%)	2 (0)	12 (0)		2 (1)	4 (0)	
Methionine (MET), median (IQR)	20.00 (17.00-24.00)	21.00 (17.00-24.00)	0.15	19.00 (17.00-22.75)	19.00 (16.00-22.00)	0.12
Missing, n (%)	3 (0)	15 (0)		2 (1)	4 (0)	
Ornithine (ORN), median (IQR)	65.00 (54.00-78.00)	66.00 (55.00-79.00)	0.13	66.00 (55.00-81.00)	66.00 (55.25-81.75)	0.93
Missing, n (%)	1 (0)	14 (0)		2 (1)	4 (0)	
Phenylalanine (PHE), median (IQR)	49.00 (42.00-56.00)	50.00 (44.00-57.00)	<0.001*	48.50 (44.00-55.00)	44.00 (49.00-55.00)	0.79
Missing, n (%)	1 (0)	20 (0)		2 (1)	6 (0)	

Succinylacetone (SUAC), median (IQR)	0.47 (0.38-0.59)	0.49 (0.40-0.60)	<0.001*	0.40 (0.35-0.47)	0.39 (0.34-0.45)	0.04*
Missing, n (%)	323 (27)	1,548 (21)		2 (1)	6 (0)	
Tyrosine (TYR), median (IQR)	73.00 (56.00-97.80)	85.00 (67.00-109.00)	<0.001*	7300 (55.25-94.00)	78.00 (62.00-99.00)	0.004*
Missing, n (%)	3 (0)	15 (0)		2 (1)	6 (0)	
Valine (VAL), median (IQR)	85.00 (72.00-99.00)	81.00 (70.00-95.00)	<0.001*	87.00 (74.00-100.00)	85.00 (72.00-101.00)	0.51
Missing, n (%)	5 (0)	18 (0)		2 (1)	6 (0)	

IQR, interquartile range.

[a]*p*-values calculated using Mann–Whitney U or Pearson χ^2, as appropriate.

*Statistically significant at $\alpha<0.05$.

--, Data suppressed (n<10).

Table S6. Associations between smoking during the third trimester of pregnancy (yes, no) and metabolite concentrations at birth by cohort.

	PRAMS (n=8600)			INSPIRE (n=1918)		
**Metabolite (umol/L)**	**Exp(β) (95% CI)**	**Adjusted exp(β) (95% CI)**	**Adjusted *p*-value[a]**	**Exp(β) (95% CI)**	**Adjusted exp(β) (95% CI)**	**Adjusted *p*-value**
Free carnitine (C0)	**1.15 (1.12, 1.17)**	**1.11 (1.08, 1.14)**	**<0.001***	**1.13 (1.07, 1.19)**	**1.08 (1.02, 1.14)**	**0.01***
**Short-chain acylcarnitines**						
Acetylcarnitine (C2)	1.04 (1.02, 1.06)	1.06 (1.04, 1.09)	<0.001*	1.02 (0.97, 1.07)	1.04 (0.98, 1.09)	0.19
Propionylcarnitine (C3)	1.03 (1.01, 1.06)	1.00 (0.98, 1.03)	0.98	N/A	N/A	N/A
Butyrylcarnitine + Isobutyrylcarnitine (C4)	1.00 (0.98, 1.02)	1.01 (0.99, 1.04)	0.56	N/A	N/A	N/A
Isovalerylcarnitine + Methylbutyrylcarnitine (C5)	1.07 (1.04, 1.09)	1.06 (1.04, 1.08)	<0.001*	1.05 (1.00, 1.11)	1.04 (0.99, 1.09)	0.17
Tiglylcarnitine (C5:1)[b]	0.98 (0.83, 1.14)	0.96 (0.80, 1.14)	0.75	N/A	N/A	N/A
**Medium-chain acylcarnitines**						
Hexanoylcarnitine (C6)	1.01 (0.99, 1.03)	1.04 (1.02, 1.07)	0.004*	0.99 (0.94, 1.04)	1.05 (0.99, 1.10)	0.09
Methylglutarylcarnitine (C6-DC)	0.99 (0.97, 1.01)	1.00 (0.97, 1.02)	0.75	N/A	N/A	N/A
Octanoylcarnitine (C8)	0.98 (0.96, 1.01)	1.03 (1.00, 1.05)	0.08	N/A	N/A	N/A
Decanoylcarnitine (C10)	0.93 (0.90, 0.95)	1.00 (0.97, 1.03)	0.90	N/A	N/A	N/A
Decenoylcarnitine (C10:1)	1.01 (0.99, 1.03)	1.02 (0.99, 1.04)	0.27	N/A	N/A	N/A
Decadienoylcarnitine (C10:2)[b]	1.33 (1.14, 1.56)	1.24 (1.04, 1.48)	0.04*	1.02 (0.61, 1.71)	0.97 (0.55, 1.72)	0.92
**Long-chain acylcarnitines**						
Tetradecanoylcarnitine (C14)	1.00 (0.98, 1.02)	1.03 (1.01, 1.05)	0.02*	0.95 (0.91, 1.00)	1.02 (0.97, 1.07)	0.57
Tetradecenoylcarnitine (C14:1)	0.90 (0.88, 0.93)	0.96 (0.94, 0.99)	0.02*	0.84 (0.79, 0.90)	0.95 (0.89, 1.01)	0.10

3-Hydroxytetradec-anoylcarnitine (C14-OH)[b]	0.73 (0.65, 0.82)	0.91 (0.80, 1.04)	0.29	N/A	N/A	N/A
Palmitoylcarnitine (C16)	0.97 (0.95, 0.99)	1.00 (0.98, 1.02)	0.96	N/A	N/A	N/A
Palmitoleylcarnitine (C16:1)	0.96 (0.94, 0.98)	0.98 (0.96, 1.01)	0.32	N/A	N/A	N/A
3-Hydroxypalmi-toylcarnitine (C16-OH)[b]	0.75 (0.67, 0.85)	0.97 (0.86, 1.11)	0.76	N/A	N/A	N/A
Stearoylcarnitine (C18)	1.03 (1.01, 1.05)	1.06 (1.03, 1.08)	<0.001*	0.99 (0.94, 1.03)	1.01 (0.96, 1.06)	0.83
Oleoylcarnitine (C18:1)	1.03 (1.01, 1.05)	1.03 (1.01, 1.06)	0.006*	1.00 (0.95, 1.04)	0.98 (0.94, 1.04)	0.53
Linoleoylcarnitine (C18:2)	1.27 (1.24, 1.30)	1.17 (1.14, 1.20)	<0.001*	1.22 (1.14, 1.30)	1.04 (0.97, 1.11)	0.26
3-Hydroxyoleoylcarnitine (C18:1-OH)[b]	0.93 (0.82, 1.05)	1.04 (0.91, 1.19)	0.75	N/A	N/A	N/A
**Amino Acids**						
Arginine (ARG)	0.98 (0.94, 1.01)	1.01 (0.97, 1.05)	0.75	N/A	N/A	N/A
Argininosuccinate (ASA)	1.00 (0.96, 1.04)	0.99 (0.94, 1.03)	0.74	N/A	N/A	N/A
Citrulline (CIT)	0.94 (0.93, 0.96)	0.97 (0.96, 0.99)	0.02*	0.96 (0.93, 1.00)	1.00 (0.96, 1.04)	0.87
Glycine (GLY)	**1.00 (0.99, 1.02)**	**1.03 (1.01, 1.04)**	**0.006***	**1.02 (0.98, 1.05)**	**1.05 (1.01, 1.09)**	**0.006***
Leucine (LEU)	**1.05 (1.04, 1.07)**	**1.04 (1.03, 1.06)**	**<0.001***	**1.05 (1.02, 1.09)**	**1.05 (1.01, 1.09)**	**0.007***
Methionine (MET)	0.99 (0.98, 1.01)	1.00 (0.98, 1.01)	0.75	N/A	N/A	N/A
Ornithine (ORN)	0.99 (0.97, 1.01)	1.00 (0.99, 1.02)	0.75	N/A	N/A	N/A
Phenylalanine (PHE)	0.97 (0.96, 0.99)	0.99 (0.98, 1.01)	0.40	N/A	N/A	N/A
Succinylacetone (SUAC)	0.97 (0.95, 0.99)	0.98 (0.95, 1.00)	0.12	N/A	N/A	N/A
Tyrosine (TYR)	0.87 (0.85, 0.89)	0.93 (0.91, 0.95)	<0.001*	0.93 (0.89, 0.98)	0.99 (0.94, 1.04)	0.72
Valine (VAL)	1.03 (1.02, 1.05)	1.04 (1.03, 1.06)	<0.001*	1.01 (0.98, 1.05)	1.04 (1.00, 1.08)	0.05

CI, confidence interval.
[a]False discovery rate corrected.
[b]Effect expressed as odds ratio or adjusted odds ratio and 95% CI.
*Statistically significant at $\alpha<0.05$.
N/A, not applicable. Association was not explored as smoking during the third trimester of pregnancy was not significantly associated with metabolite in the discovery cohort (PRAMS).

Fold change increases in the medians (exp[β]) were calculated for metabolites with continuous distributions (log-transformed) using linear regression. Odds ratios were calculated for metabolites using proportional odds regression. Regression models were adjusted for maternal age at delivery, maternal race and ethnicity, type of health insurance, maternal pre-pregnancy BMI, and ever breastfed.

Figure S6. **Dose-response curves for associations between number of cigarettes smoked during the third trimester of pregnancy among women who smoked during this time and newborn concentrations of metabolites that were statistically significant in the primary analysis.**

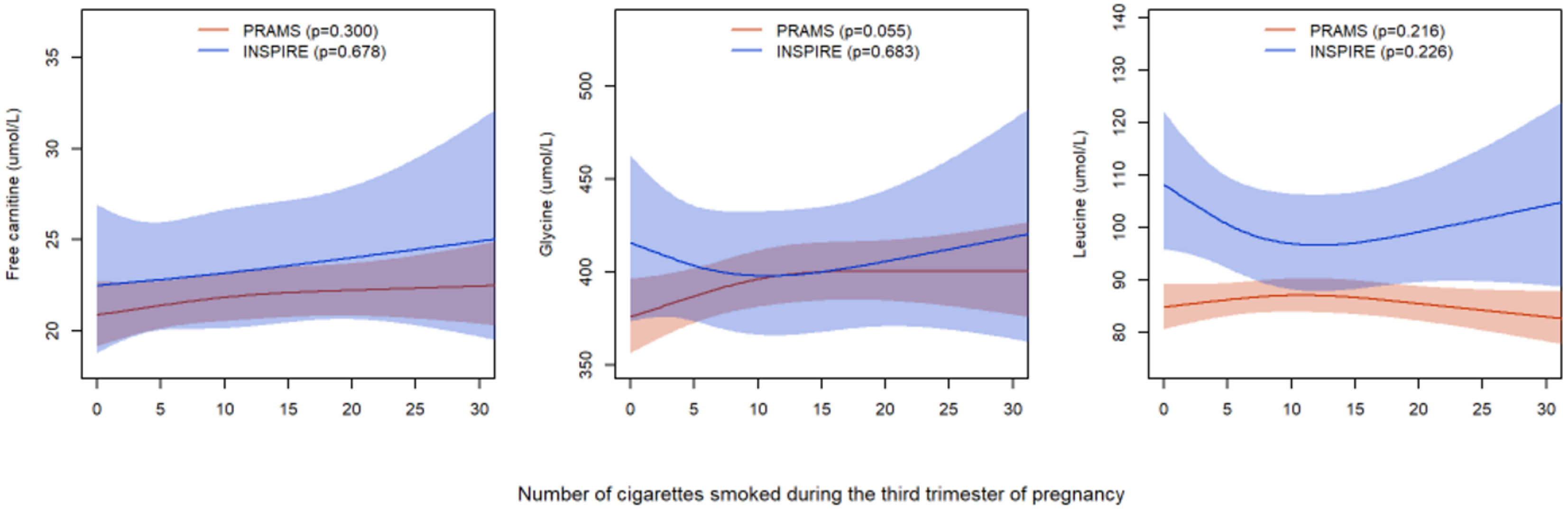

Fold change increases in the medians (exp[β]) of log-transformed metabolites were calculated using linear regression. Regression models were adjusted for maternal age at delivery, maternal race and ethnicity, type of health insurance, maternal pre-pregnancy BMI, and ever breastfed. Restricted cubic splines were used for number of cigarettes smoked during the third trimester of pregnancy, including three knots located at the 10th, 50th, and 90th percentiles of the distribution of number of daily cigarettes used.

Table S7. Newborn screening metabolite concentrations by pattern of smoking during pregnancy and cohort.

	PRAMS				INSPIRE			
**Metabolite**	**Non-smoker**	**Quitter**	**Continued smoker**	***p*-value[a]**	**Non-smoker**	**Quitter**	**Continued smoker**	***p*-value[a]**
**Sample size, n (%)**	**6,782 (79)**	**598 (7)**	**1,199 (14)**		**1,383 (72)**	**294 (15)**	**237 (12)**	
Free carnitine (C0), median (IQR)	19.86 (15.17-26.53)	20.16 (15.60-25.70)	22.69 (17.38-30.65)	<0.001*	17.52 (13.83-22.52)	18.86 (14.29-23.98)	20.27 (15.88-25.24)	<0.001*
Missing, n (%)	17 (0)	1 (0)	2 (0)		6 (0)	0 (0)	2 (1)	
Glycine (GLY), median (IQR)	385.00 (330.00-450.48)	386.00 (326.00-447.00)	388.50 (328.00-462.00)	0.65	384.00 (329.50-444.00)	374.00 (317.50-439.75)	391.00 (338.50-446.00)	0.19
Missing, n (%)	15 (0)	0 (0)	3 (0)		4 (0)	0 (0)	2 (1)	
Leucine (LEU), median (IQR)	81.00 (70.00-94.00)	82.00 (71.00-95.00)	86.00 (73.00-100.00)	<0.001*	89.00 (76.00-104.00)	89.00 (77.00-103.00)	94.00 (82.50-109.00)	0.004*
Missing, n (%)	12 (0)	0 (0)	2 (0)		4 (0)	0 (0)	2 (1)	

IQR—interquartile range.
[a]*p*-values calculated using Kruskal–Wallis or Pearson χ^2, as appropriate.
*Statistically significant at α<0.05.

Figure S7. Dose-response curves for associations between number of cigarettes smoked during the third trimester of pregnancy among women who smoked and newborn concentrations of metabolites that were statistically significant in the primary analysis after multiple imputation.

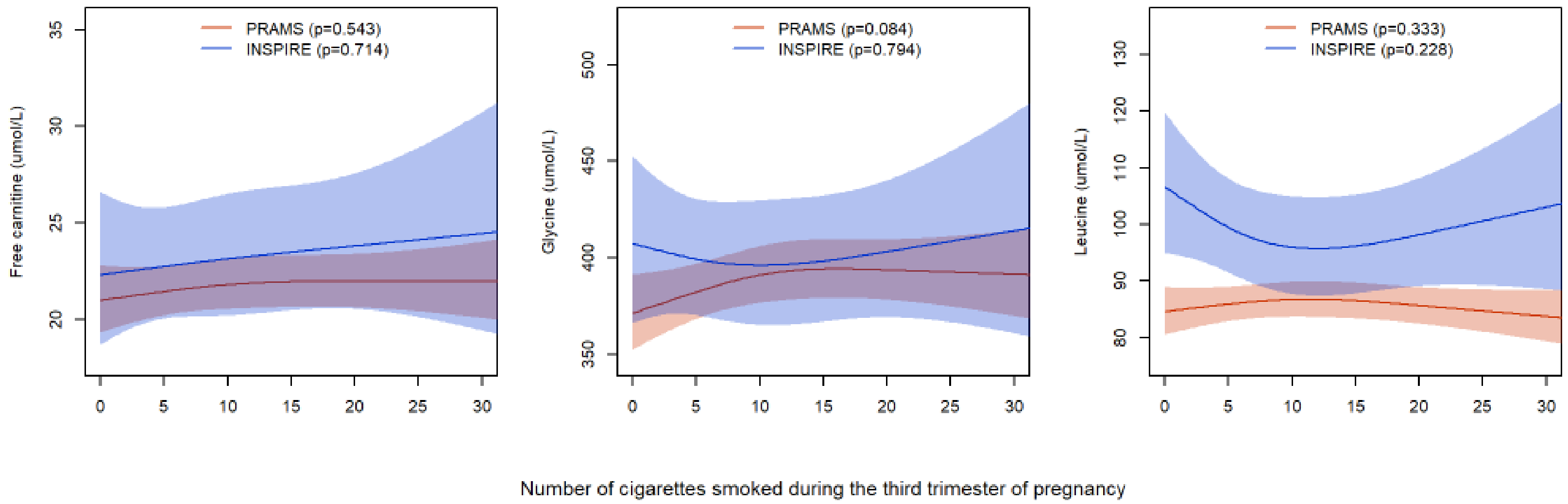

Fold change increases in the medians (exp[β]) of log-transformed metabolites were calculated using linear regression. Regression models were adjusted for maternal age at delivery, maternal race and ethnicity, type of health insurance, maternal pre-pregnancy BMI, and ever breastfed. Restricted cubic splines were used for number of cigarettes smoked during the third trimester of pregnancy, including three knots located at the 10th, 50th, and 90th percentiles of the distribution of number of daily cigarettes used.

Figure S8. Associations between smoking status before and during pregnancy and free carnitine (C0), glycine (GLY), and leucine (LEU) concentrations at birth by cohort with and without (i.e., complete case) multiple imputation.

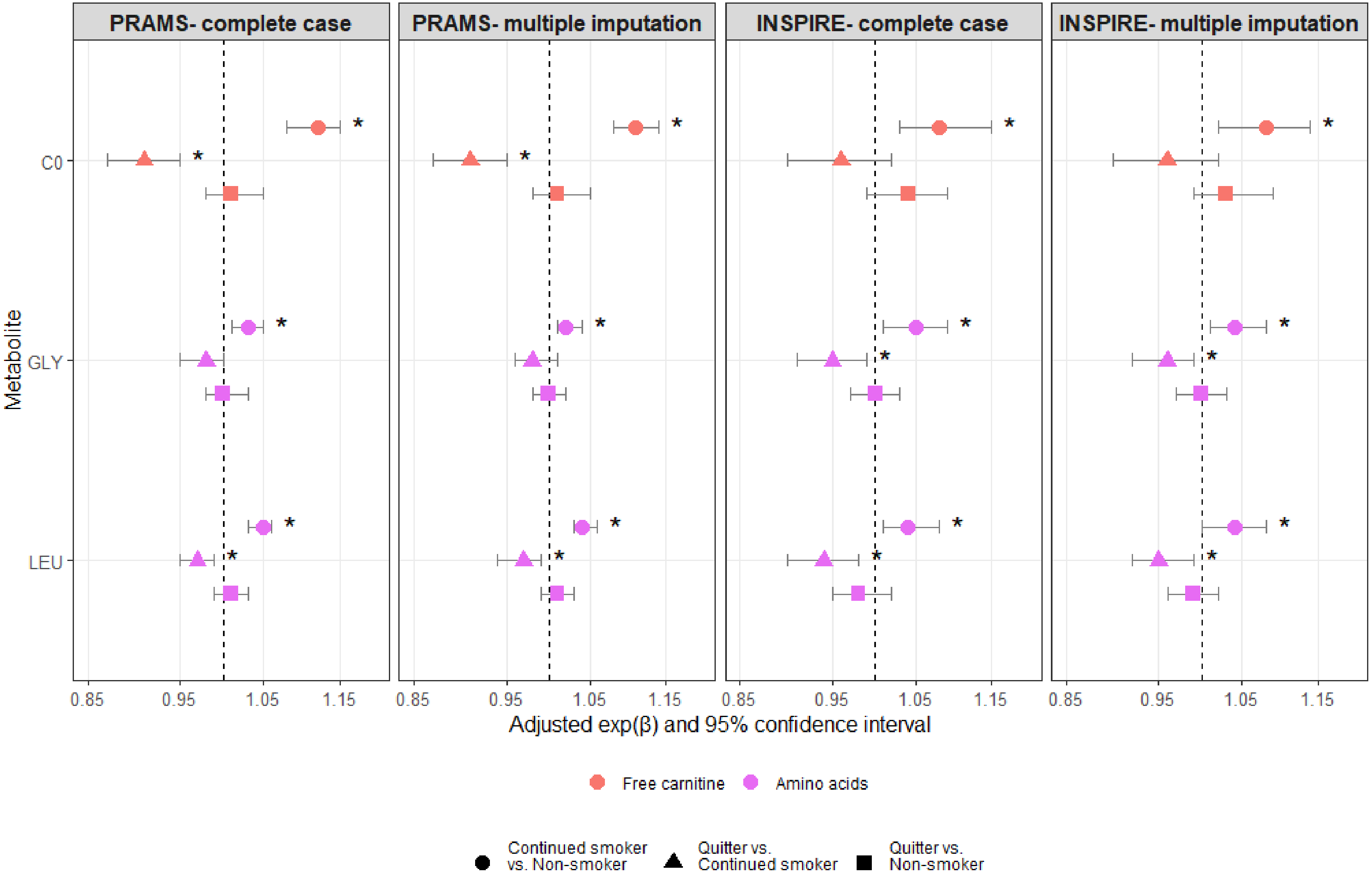

Fold change increases in the medians (exp[β]) were calculated for metabolites with continuous distributions (log-transformed) using linear regression. Regression models were adjusted for maternal age at delivery, maternal race and ethnicity, type of health insurance, maternal pre-pregnancy BMI, and ever breastfed. *Adjusted *p*-value significant at $\alpha<0.05$.
